# Socially Distanced Neonatal Resuscitation Program (NRP): A Technical Report on How to Teach NRP Courses During the COVID-19 Pandemic

**DOI:** 10.7759/cureus.10959

**Published:** 2020-10-15

**Authors:** Kristy Robinson, Hai-Yen Tang, Erika Metzenberg, Jenifer Peterson, Rachel Umoren, Taylor Sawyer

**Affiliations:** 1 Outreach Education, Seattle Children's Hospital, Seattle, USA; 2 Critical Care, Seattle Children's Hospital, Seattle, USA; 3 Pediatrics, University of Washington School of Medicine, Seattle, USA; 4 Pediatrics, Seattle Children's Hospital, Seattle, USA

**Keywords:** neonatal resuscitation program, physical distancing, simulation

## Abstract

In this technical report, we describe a method for teaching the Neonatal Resuscitation Program (NRP) courses while also maintaining social distancing during the COVID-19 pandemic: a method we call ‘Socially Distanced NRP.’ The unique aspects of Socially Distanced NRP courses include small class sizes, keeping one group of students and their instructors together throughout the course, and creating socially distanced stations where students complete the performance skills, integrated skills, and simulation and debriefing parts of the NRP course. The four socially distanced stations include airway, chest compressions, umbilical venous catheter placement, and team leader. Feedback from 79 NRP students showed no difference in overall course rating between Socially Distanced NRP and standard NRP courses. No cases of COVID-19 transmission were identified in the Socially Distanced NRP courses. We believe that Socially Distanced NRP is a safe and effective way to provide mandatory NRP training during the COVID-19 pandemic.

## Introduction

The Neonatal Resuscitation Program (NRP) is a simulation-based educational program that teaches healthcare providers the cognitive, technical, and behavioral skills required to perform a neonatal resuscitation [1]. The NRP Provider course traditionally uses an in-person instructor-facilitated practice that requires students to work together as a team in close proximity during simulated delivery room resuscitation [1]. The in-person course components include performance skills stations, integrated skills stations, and simulation and debriefing [1]. The COVID-19 pandemic has resulted in a broad array of restrictions and precautions, including the use of personal protective equipment (PPE) and the need for social distancing. These restrictions have raised many questions about how to conduct effective NRP courses during the COVID-19 pandemic.

The NRP has published strategies for teaching NRP courses during COVID-19 [2]. Suggested strategies include screening instructors and learners for COVID-19 symptoms, wearing PPE, sanitizing manikins and equipment, and using as large a space as possible to maintain social distancing. However, no detailed guidance has been published on conducting an NRP course while also maintaining social distancing.

In this technical report, we describe a method for teaching NRP courses while also maintaining social distancing: a method we call ‘Socially Distanced NRP.’ The methods described here were developed initially to provide NRP courses to a group of 45 first-year pediatric residents who were required to take NRP during the COVID-19 pandemic. After successfully using the framework for our first-year residents, we have adopted this method for all our NRP courses during the COVID-19 pandemic.

## Technical report

Instructor and student organization

A basic premise of a Socially Distanced NRP class is to keep the class size small. We aim for no more than four students to each instructor. Students and instructors are divided into groups. Each student and instructor group is kept together throughout the course and stay in a single classroom. The length of the Socially Distanced NRP class is the same as the standard NRP course, at approximately four hours. For large classes, multiple separate classrooms are needed. 

Room setup

**Figure 1:**
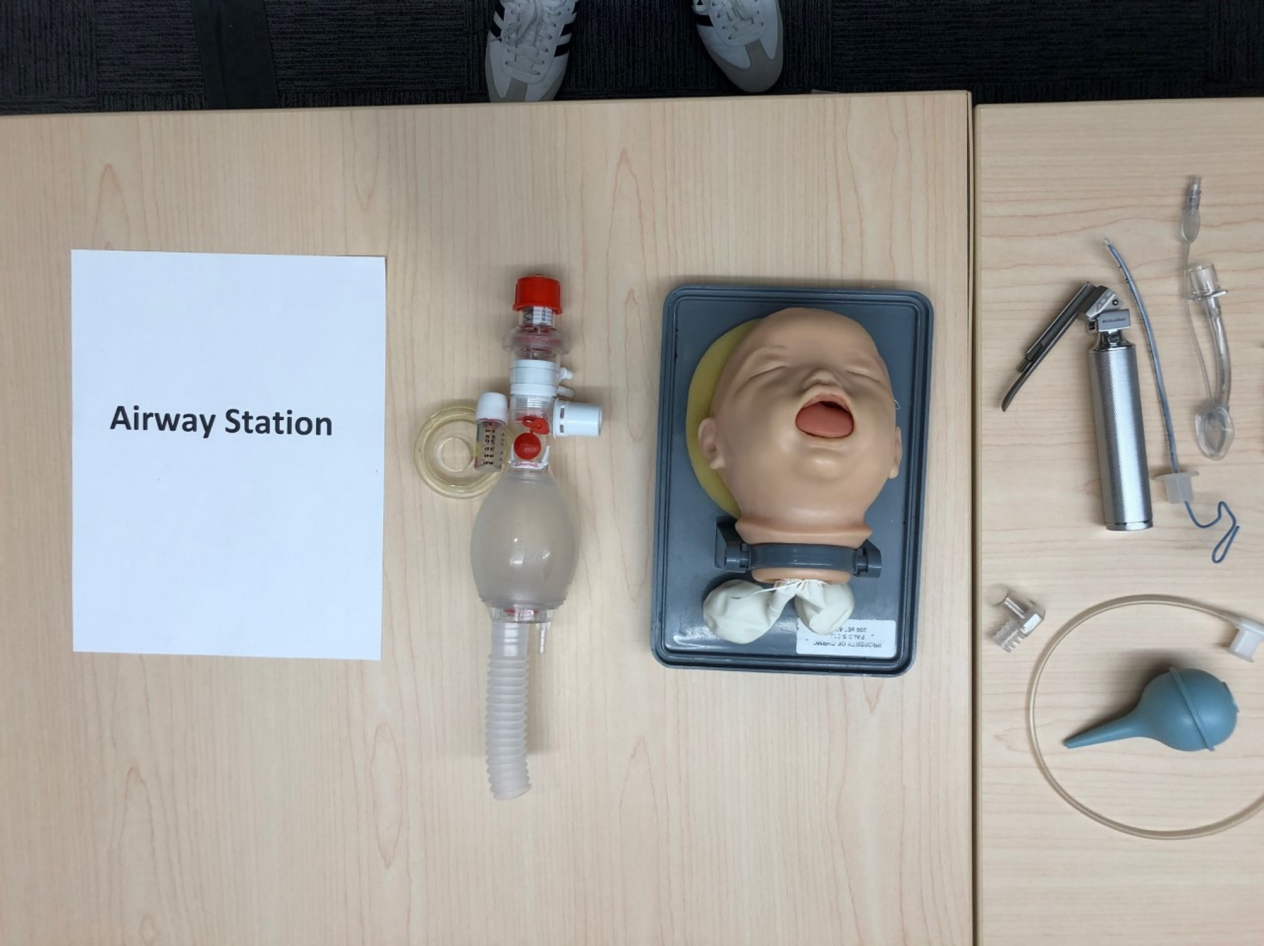
Airway Station Supplies include neonatal intubation head, laryngoscope and blades, endotracheal tubes and stylets, laryngeal masks, 5ml syringe, CO2 detector, bulb syringe, suction catheter, positive pressure ventilation (PPV) device, and face masks.

**Figure 2:**
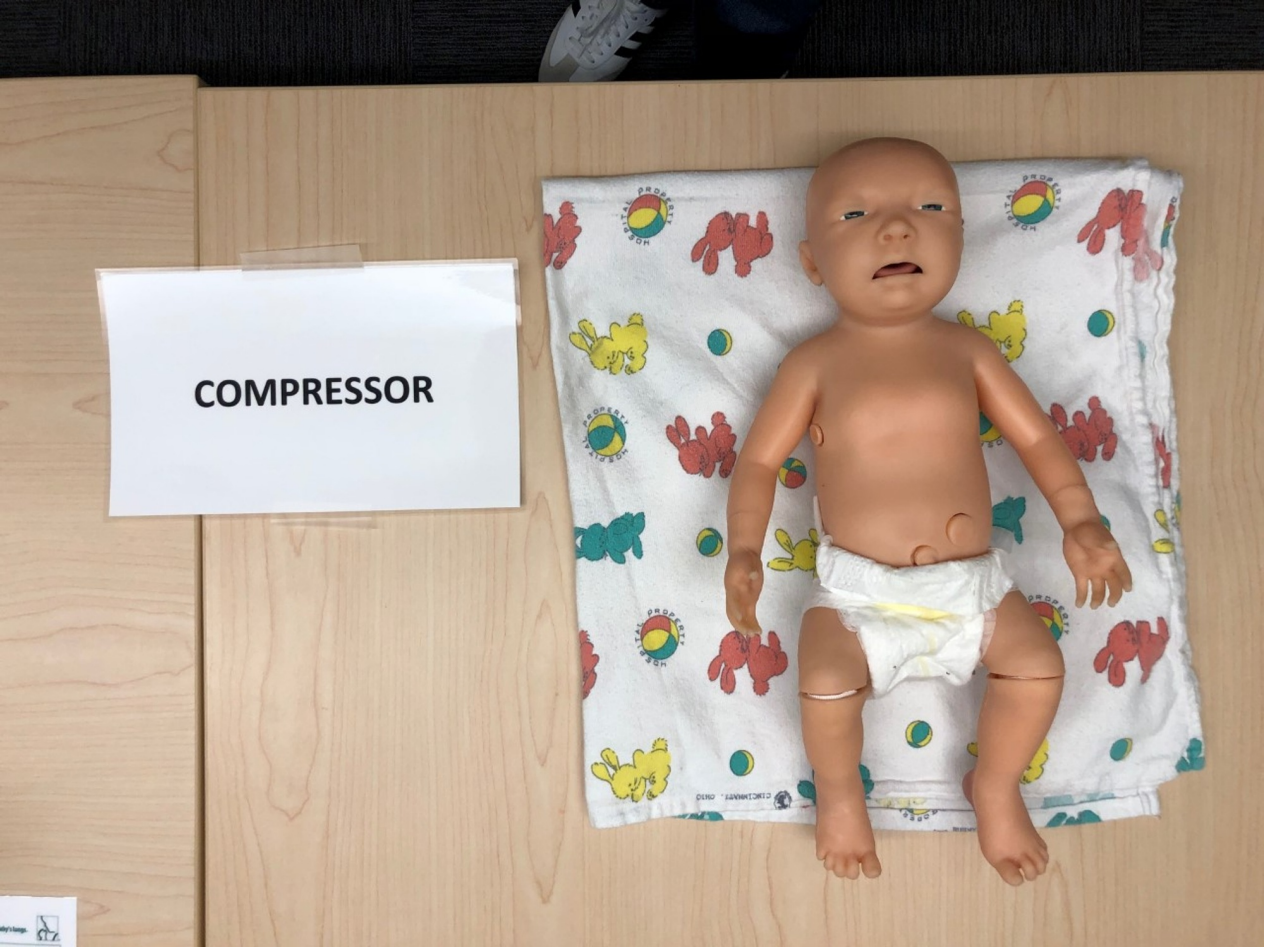
Chest Compression Station Supplies include neonatal manikin, blanket, stethoscope, ECG leads, pulse-oximeter, and iPad used as a monitor with Apgar timer (monitoring equipment not shown in photo).

**Figure 3:**
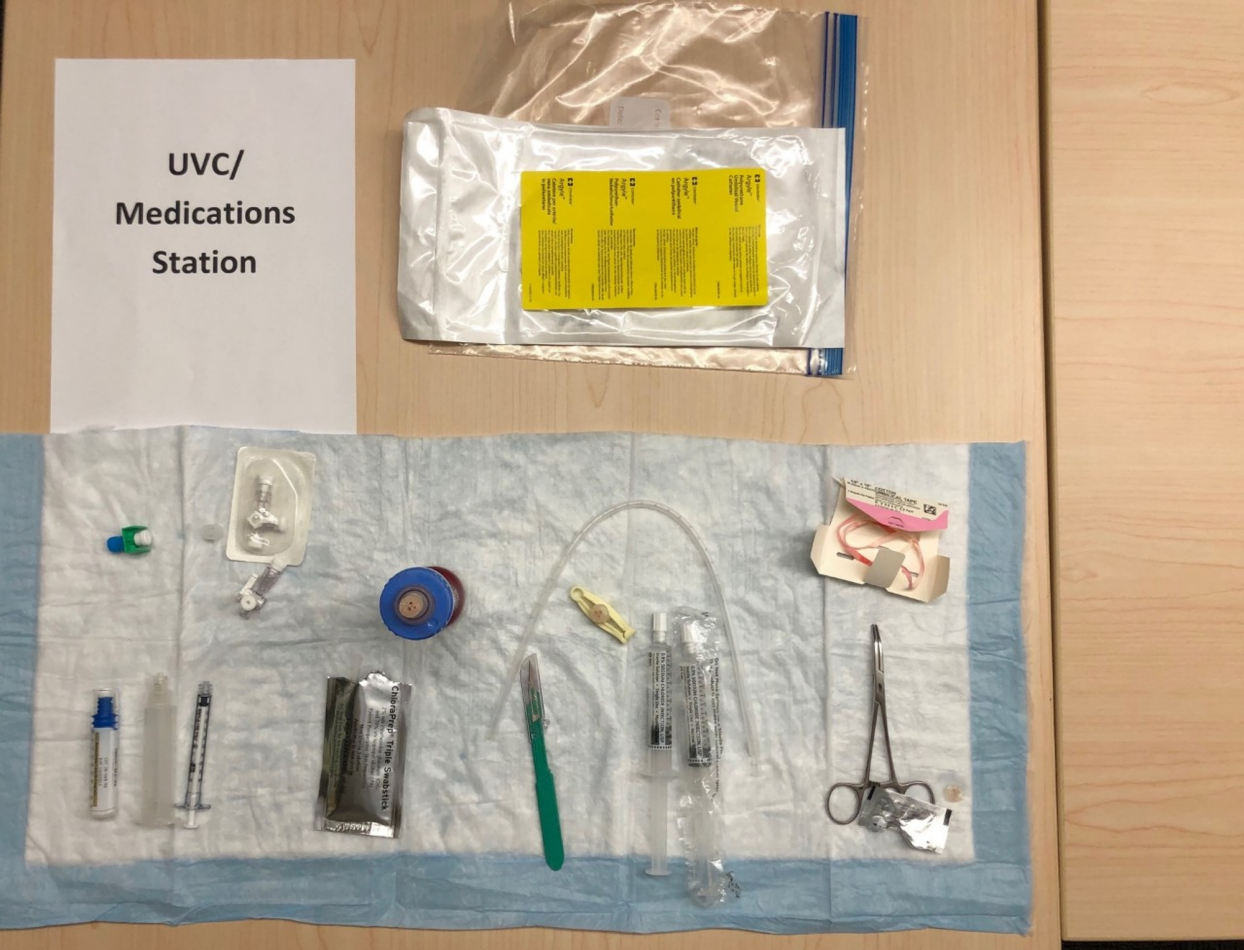
Umbilical Venous Catheter (UVC) Station Supplies include 3.5 fr umbilical catheter, 3-way stop-cocks, a transfer device, one box of epinephrine, 1ml syringe, chloraprep swabs, scalpel, 10ml flushes, hemostat, umbilical tape, and a UVC insertion trainer.

Rooms are set up to encourage social distancing. Each classroom has marks on the floor, indicating where participants should stand during the performance skills stations, integrated skills station, and simulation and debriefing. Each marked area was approximately six feet apart and corresponded to a single neonatal resuscitation team member’s role. We included four separate skills stations in our Socially Distanced NRP classes: airway, chest compressions, umbilical venous catheter (UVC) placement, and team leader. The airway station is set up with a neonatal intubation head and airway supplies. (Figure 1) The chest compression station includes a neonatal manikin and monitoring equipment. (Figure 2) The UVC placement station includes an umbilical catheter trainer, UVC supplies, and epinephrine. (Figure 3) The team leader station has a copy of the NRP algorithm and other reference materials.

 

 

In addition to the changes in group size and class structure noted above, several additional precautions were used to decrease the risk of COVID-19 amongst students and instructors. In compliance with our institution’s guidelines [3], All instructors and students were screened for illness before entering the facility. Each person was given directions to stay in their assigned room, except to use the restroom. Masks were always worn. Hand hygiene was encouraged using gloves and frequent use of hand sanitizer. All equipment was wiped down with alcohol-based wipes between uses. Laminated posters of the COVID-19 prevention measures were displayed in each classroom for reference.

Performance skills and integrated skills stations

**Figure 4:**
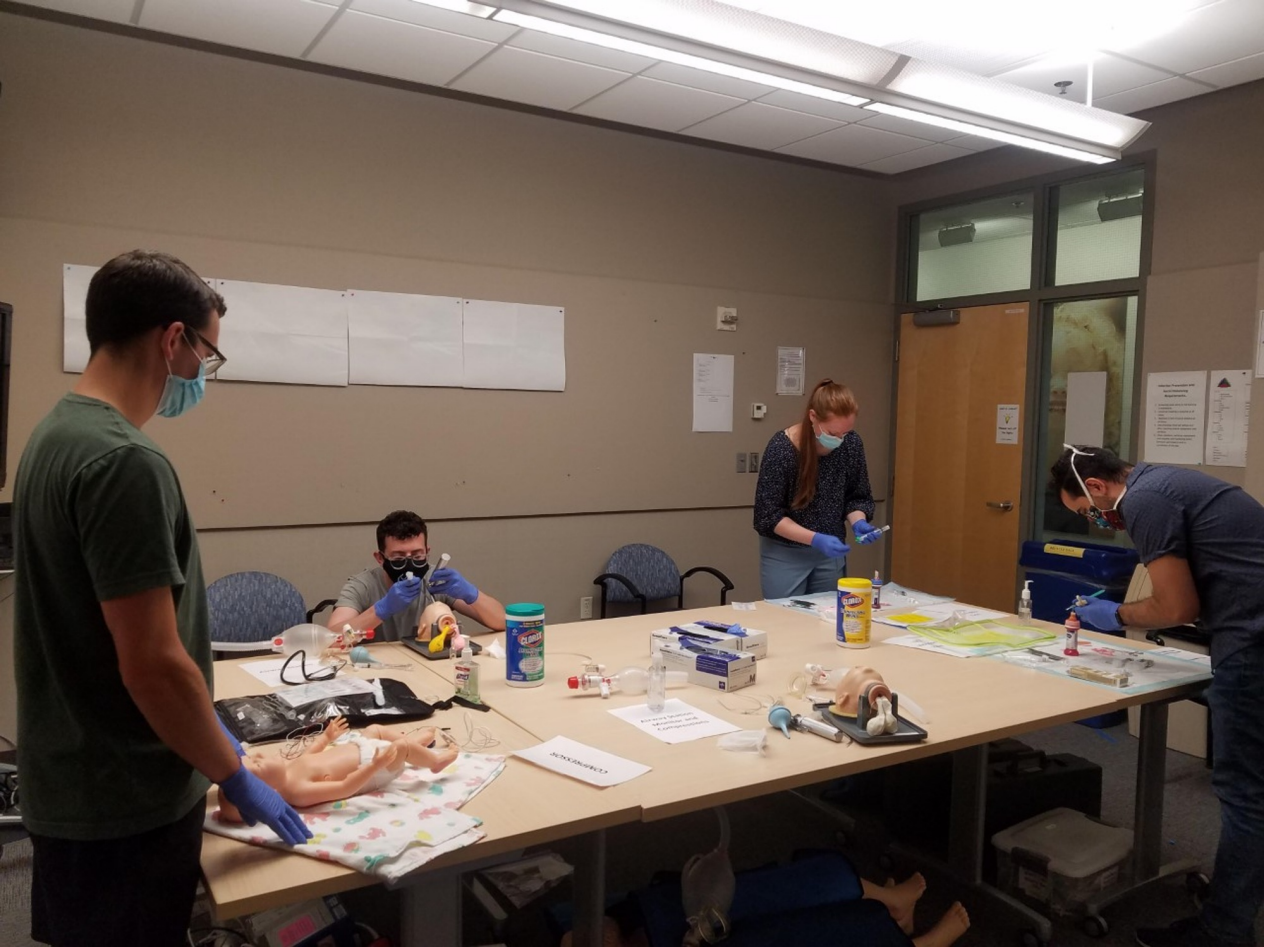
Performance Skills Stations The two students on the left are working on airway skills. The two students on the right are working on UVC placement skills.

The Socially Distanced NRP course includes four performance skills stations used for reviewing, practicing, and performing neonatal resuscitation skills. (Figure 4) The performance skills stations included airway skills, chest compressions, umbilical venous catheter (UVC) placement, and team leader - as noted above. Preparation for delivery and initial steps of resuscitation was performed at the team leader station, where we had each student say out loud how they would prepare equipment and perform the initial steps of resuscitation. To allow the instructor to provide adequate coaching, the performance skills are taught sequentially in the following order: initial steps, airway skills, chest compressions, and UVC placement. Students rotate through each station and receive feedback from the instructor. 

In addition to the performance skills stations, the Socially Distanced NRP course also included an integrated skills station used to evaluate a student’s ability to correctly incorporate all relevant NRP resuscitation skills into a scenario without instructor assistance. In the Socially Distanced NRP course, each student rotated through each of the four skills stations noted above, and their skills were evaluated in order to complete the integrated skills station. Only after rotating at each station and proving their ability to complete all skills were the students allowed to progress to simulation and debriefing.

Simulation and debriefing

**Figure 5:**
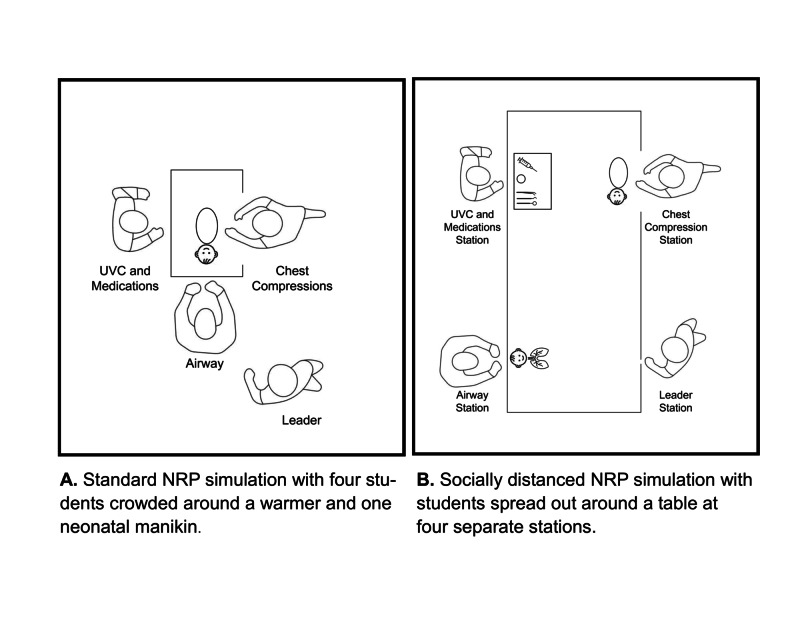
Comparison of standard NRP simulation (A) and Socially Distanced NRP simulation (B)

The simulation and debriefing portion of the NRP course is perhaps the hardest portion in which to maintain safe social distancing. During the simulation and debriefing part of the Socially Distanced NRP course the students work together to perform a simulated neonatal resuscitation, but each team member is socially distanced by standing at one of the four stations noted above. Dividing the team members by role and task this way aligns well with the practice of task-oriented role assignment (TORA) recently described by Litke-Wager, et al. [4]. See Figure 5 for a visual comparison of a standard NRP course to a Socially Distanced NRP course.

**Figure 6:**
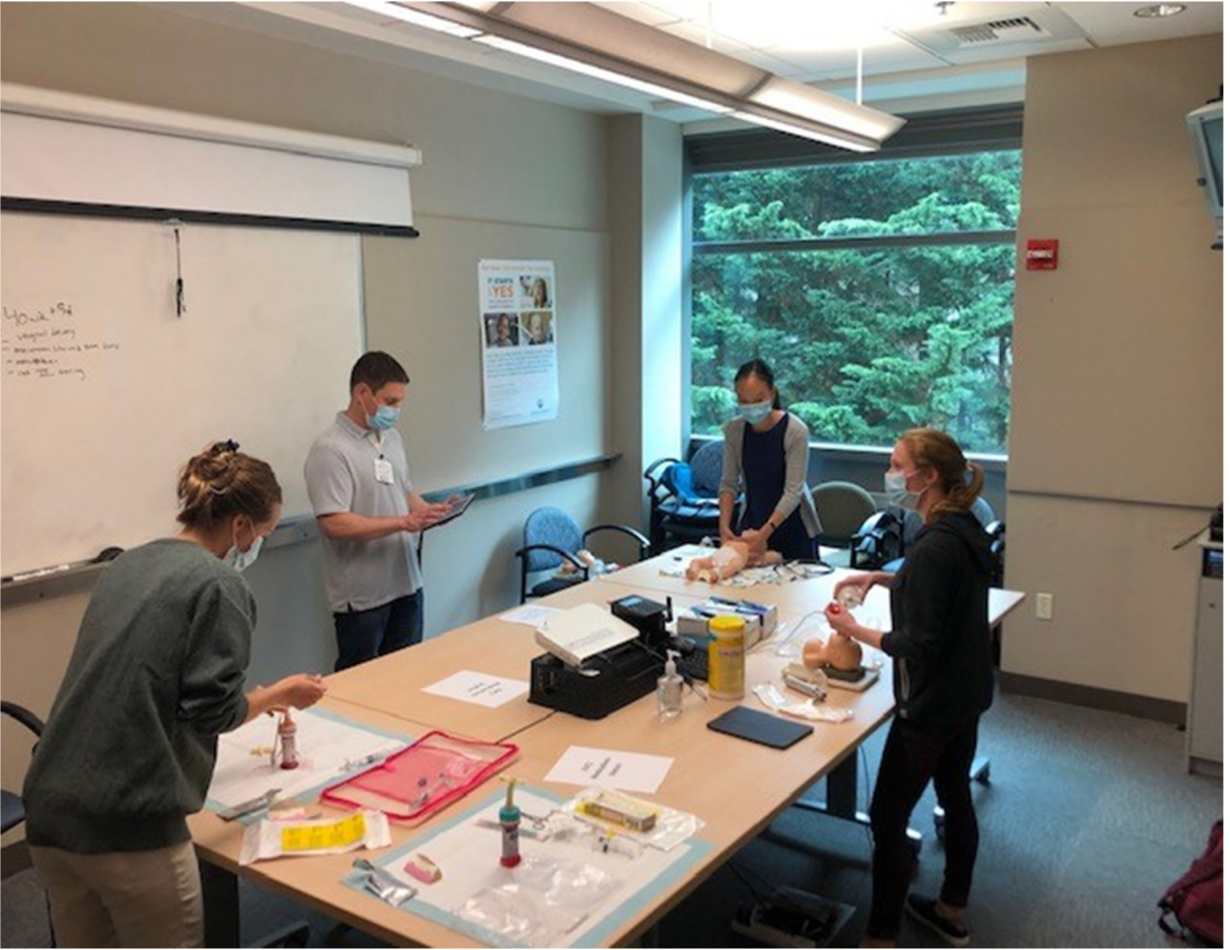
Simulation and Debriefing The student near the whiteboard is the team leader. The student on the far right is providing breaths through an endotracheal tube. The student in front of the window is performing chest compressions. The student on the far left is placing a UVC. While physically separated, all students are working together in real-time.

The simulation starts with a scenario lead-in by the instructor and a brief preparation period by the team. The team leader directs preparation for the delivery and initial steps of resuscitation. Once the simulation starts, the students at each station perform the resuscitation skills relevant to their respective stations with the coordination of all activities by the team leader. When positive pressure ventilation or intubation is needed, the airway station student uses the intubation head to perform the skill(s). If chest compressions are needed, the student at the chest compression station performs that skill. Coordination between the airway station and chest compression station is needed to ensure the proper 3:1 compression: ventilation ratio. Even though the students are physically separated, they can work together in a coordinated manner. When UVC placement is needed, the student at that station uses the UVC supplies to place the line and administers epinephrine and/or fluids. After each simulation, a debriefing is conducted. See Figure 6 for a photograph of simulation and debriefing during a Socially Distanced NRP course. After the debriefing, students rotate positions to participate in the next simulation scenario. Once all four students rotate through all four roles, the simulation and debriefing part of the course is complete.

## Discussion

In this technical report, we describe a method for conducting Socially Distanced NRP courses during the COVID-19 pandemic. Changes made to the standard NRP course included limiting the student group size to four, having students stay in a single classroom, and physically separating students by using predefined skills stations. Each skill station is associated with an NRP team member’s role. Students rotated stations to complete the performance skills stations, integrated skills station, and simulation and debriefing parts of the NRP course.

The need to create Socially Distanced NRP courses arose in March 2020 when the prevalence of COVID-19 cases in our region resulted in a state-wide stay at home order. This order resulted in the cancellation of all in-person resuscitation classes provided by our organization. As our organization began to return to more normal functioning in June 2020, we welcomed an incoming pediatric resident class. To complete mandatory NRP training of these new residents, it became critical to develop a novel NRP course design that was both safe and effective. 

The Socially Distanced NRP course made it safe to conduct NRP training during the COVID-19 pandemic. However, the changes to the course may have impacted the student experiences. We compared post-course evaluation data from standard NRP course and Socially Distanced NRP courses to examine student experiences. The comparison groups included 44 first-year pediatric residents who completed a standard NRP course in 2019 and 35 first-year pediatric residents who completed a Socially Distanced NRP in 2020. The results are provided in Table 1. The overall course ratings were not significantly different between the two years. In both years, 100% of students reported that the environment felt safe and supportive of learning needs. Students did report lower levels of satisfaction with simulation and debriefing in the Socially Distanced NRP. Students did report lower levels of agreement that the skills review and practice component of the course was useful and lower satisfaction with simulation and debriefing experience in the Socially Distanced NRP.

**Table 1:** Post-Course Evaluation Comparison of Socially Distanced NRP to Standard NRP Course

Question	Standard NRP (n = 44)	Socially Distanced NRP (n = 35)	P value
Overall course rating, mean (SD)*	4.9 (0.3)	4.7 (0.5)	0.09
The skills review and practice component of the course was useful**	4.9 (0.2)	4.6 (0.5)	0.001
Satisfaction with simulation and debriefing experience*	4.8 (0.4)	4.6 (0.5)	0.02
The environment felt safe and supportive of my learning needs^†^, n (%)	44 (100)	35 (100)	NS
*Scale from 1 (‘Very Dissatisfied’) to 5 (‘Very Satisfied’) ** Scale from 1 (‘Strongly Disagree’) to 5 (‘Strongly Agree’) ^† ^Answered ‘Yes’ or ‘No’			

There are some limitations to the Socially Distanced NRP course described here. In each course, there were situations when it was impossible to maintain the goal of six-foot social distancing. For example, when an instructor had to provide hands-on training and corrective feedback at the performance skills stations. In those circumstances, we tried to limit the duration of proximity to less than 10 minutes. Additionally, Socially Distanced NRP course may require more physical space than a standard NRP course. Because each small group was confined to its own classroom finding a location with adequate space may be a challenge. In situations where separate rooms are not available, student-instructor teams may be separated using physical barriers such as plexiglass shields or other room dividers. These limitations should be kept in mind when considering doing Socially Distanced NRP courses. Overall, however, we believe our Socially Distanced NRP courses are a safe and effective solution to teaching NRP during the COVID-19 pandemic. No cases of COVID-19 transmission were identified during any of our Socially Distanced NRP courses.

## Conclusions

In this technical report, we described a method for teaching NRP courses while also maintaining social distancing during the COVID-19 pandemic. The unique aspects of Socially Distanced NRP courses include small class sizes, keeping one group of students and their instructors together throughout the course, and creating socially distanced stations where students complete the performance skills, integrated skills, and simulation and debriefing parts of the NRP course. The four socially distanced stations include airway, chest compressions, umbilical venous catheter placement, and team leader. Feedback from 79 NRP students showed no difference in overall course rating between Socially Distanced NRP and standard NRP courses. No cases of COVID-19 transmission were identified in the Socially Distanced NRP courses. We believe that Socially Distanced NRP is a safe and effective way to provide mandatory NRP training during the COVID-19 pandemic.

## References

[1] Sawyer T, Ades A, Ernst K, Colby C (2016). Simulation and the neonatal resuscitation program 7th edition curriculum. NeoReviews.

[2] (2020). The NRP provider course: strategies for teaching during COVID-19. https://downloads.aap.org/AAP/PDF/NRP_Provider_Course_Strategies_Teaching_During_COVID19.pdf.

[3] (2020). Information about COVID-19 (Novel Coronavirus). https://www.seattlechildrens.org/patients-families/covid-19-novel-coronavirus/.

[4] Litke-Wager C, Delaney H, Mu T, Sawyer T (2020). Impact of task-oriented role assignment on neonatal resuscitation performance: a simulation-based randomized controlled trial. Am J Perinatol.

